# Vitiligo and Metabolic Syndrome: Systematic Review and Meta-Analysis

**DOI:** 10.2196/34772

**Published:** 2022-03-16

**Authors:** Joyce Xia, Christina Melian, William Guo, Hunya Usmani, Richard Clark, Daniel Lozeau

**Affiliations:** 1 Renaissance School of Medicine Stony Brook University Stony Brook, NY United States; 2 Department of Dermatology Renaissance School of Medicine Stony Brook University Stony Brook, NY United States; 3 Department of Biomedical Engineering Renaissance School of Medicine Stony Brook University Stony Brook, NY United States; 4 Department of Pathology Renaissance School of Medicine Stony Brook University Stony Brook, NY United States

**Keywords:** vitiligo, leukoderma, metabolic syndrome X, dysmetabolic syndrome X, insulin resistance syndrome X, syndrome X

## Abstract

**Background:**

Metabolic syndrome (MetS) has been associated with various skin conditions including vitiligo. However, the association between these 2 conditions has yet to be determined by quantitative meta-analysis.

**Objective:**

The aim of this paper was to determine the association between vitiligo and metabolic syndrome via systematic review and meta-analysis.

**Methods:**

A systematic literature search of Pubmed, Embase, Cochrane, and Web of Science was performed for all published literature prior to August 16, 2020. Case control and prospective cross-sectional studies analyzing the association between vitiligo and MetS were included in this review. The primary outcome measures include the type of vitiligo, diagnostic criteria for MetS, components of MetS (waist circumference, blood pressure, triglycerides, fasting glycemic index, and high-density lipoprotein cholesterol), low-density lipoprotein cholesterol levels, and BMI. A meta-analysis was performed to evaluate the prevalence and association of MetS in patients with vitiligo.

**Results:**

A total of 6 studies (n=734 participants) meeting eligibility criteria were included for systematic review and meta-analysis. The pooled prevalence of MetS in patients with vitiligo was (0.296, 95% CI 0.206, 0.386; *P*<.001). Patients with vitiligo were no more likely to develop MetS compared to control patients (odds ratio 1.66, 95% CI 0.83, 3.33; *P*=.01). A leave-one-out sensitivity analysis showed a significant association between MetS and vitiligo (*P*<.001). Significant elevations in fasting glycemic index (mean difference 5.35, 95% CI 2.77, 7.93; *P*<.001) and diastolic blood pressure (mean difference 1.97, 95% CI 0.02, 3.92; *P*=.05) were observed in patients with vitiligo compared to control patients.

**Conclusions:**

The association between vitiligo and metabolic syndrome carries important clinical implications. Dermatologists and other multidisciplinary team members should remain vigilant when treating this patient population in order to prevent serious cardiovascular complications that may arise as a result of metabolic disease.

## Introduction

Vitiligo is a depigmentary condition of the skin and hair follicles due to autoimmune destruction of melanocytes [1], affecting an estimated 1% of the world’s population [2]. Vitiligo lesions commonly appear on exposed areas such as the face and extremities and can increase in size and number over time, frequently causing significant psychological impact to patients’ quality of life [1,3]. Diagnosis is typically clinical and can be further subdivided into 3 major subtypes, which are nonsegmental, segmental, and unclassified [1,4]. The most common nonsegmental subtype (encompassing generalized vitiligo [4]) typically presents with a symmetric distribution and has a strong association with other autoimmune diseases, while the segmental subtype presents with a unilateral distribution and is less strongly associated with other autoimmune diseases [5]. The unclassified subtype encompasses rare variants of the disease [4]. Though the precise etiology of vitiligo remains unknown, it is hypothesized that CD4+ and CD8+ lymphocytes play a role in the pathogenesis. The involvement of cytokines such as tumor necrosis factor alpha (TNF-α), Interferon gamma (IFN-γ), interleukin (IL)-1, IL-6, IL-10, and IL-17 have also been linked to the disease [2,6]. Furthermore, patients with vitiligo and their first-degree relatives have been shown to have increased prevalence of other autoimmune conditions such as thyroid disease, type 1 diabetes mellitus, pernicious anemia, rheumatoid arthritis, Addison disease, lupus, and Guillain-Barré [1].

Metabolic disturbances are commonly seen in patients with systemic vitiligo [7]. Metabolic syndrome (MetS) is a collection of clinical findings that, when present, increases a patient’s risk of developing cardiovascular disease and type 2 diabetes [8]. Though several definitions of MetS exist, 3 of the most commonly used guidelines include the National Cholesterol Education Program **(**NCEP) Adult Treatment Panel (ATP) III criteria, the International Diabetes Federation (IDF) criteria, and the Harmonization criteria, which is a result of a joint statement released by the IDF, American Heart Association, National Heart, Lung, and Blood Institute, World Heart Federation, International Atherosclerosis Society, and International Association for the Study of Obesity in 2009 to unify ATPIII and IDF guidelines [9,10]. Regardless of the diagnostic criteria used, core features such as insulin resistance, visceral adiposity, dyslipidemia, and endothelial dysfunction are central to the development of MetS [11]. Overall, it is estimated that up to a quarter of the world population may meet MetS criteria [9]. In addition to the increased risk for cardiovascular disease and type 2 diabetes, other associations seen with MetS include fatty liver disease, hepatocellular carcinoma, chronic kidney disease, polycystic ovary syndrome, and more [12-15].

Current literature suggests a potential link between vitiligo and MetS, based on a similar pathogenesis involving proinflammatory cytokines [7]. Insulin resistance and lipid profile disturbances have demonstrated a higher prevalence in patients with vitiligo when compared to age-matched and BMI-matched control groups [16]. In fact, several articles have reported a strong association between vitiligo and both type 1 and 2 diabetes mellitus; while the association between vitiligo and type 1 diabetes is not surprising given the autoimmune nature of both conditions, the association with type 2 diabetes necessitates close surveillance for metabolic derangements [17,18]. Despite the relationship between vitiligo and type 2 diabetes mellitus, few studies have investigated the relationship between vitiligo and MetS. Of the few studies that exist, some such as that by Atas et al [19] have noted a significant correlation whereas others, such as the study by Sallam et al [20] did not note such findings. Furthermore, in a recent study of patients with nonsegmental vitiligo (n=70), a significantly higher risk of cardiovascular disease was seen in those with more chronic and severe disease or concomitant MetS. Therefore, early diagnosis and treatment of MetS in patients with vitiligo may reduce cardiovascular complications [21]. While vitiligo is typically managed by a multidisciplinary team, increased vigilance of dermatologic signs of MetS, such as acanthosis nigricans, may allow for the early detection of disease progression [22]. In this paper, we conducted a systematic review and meta-analysis to resolve the current conflicts in the literature and to analyze the association between vitiligo and MetS with an emphasis on disease prevention and early detection.

## Methods

**Figure 1:**
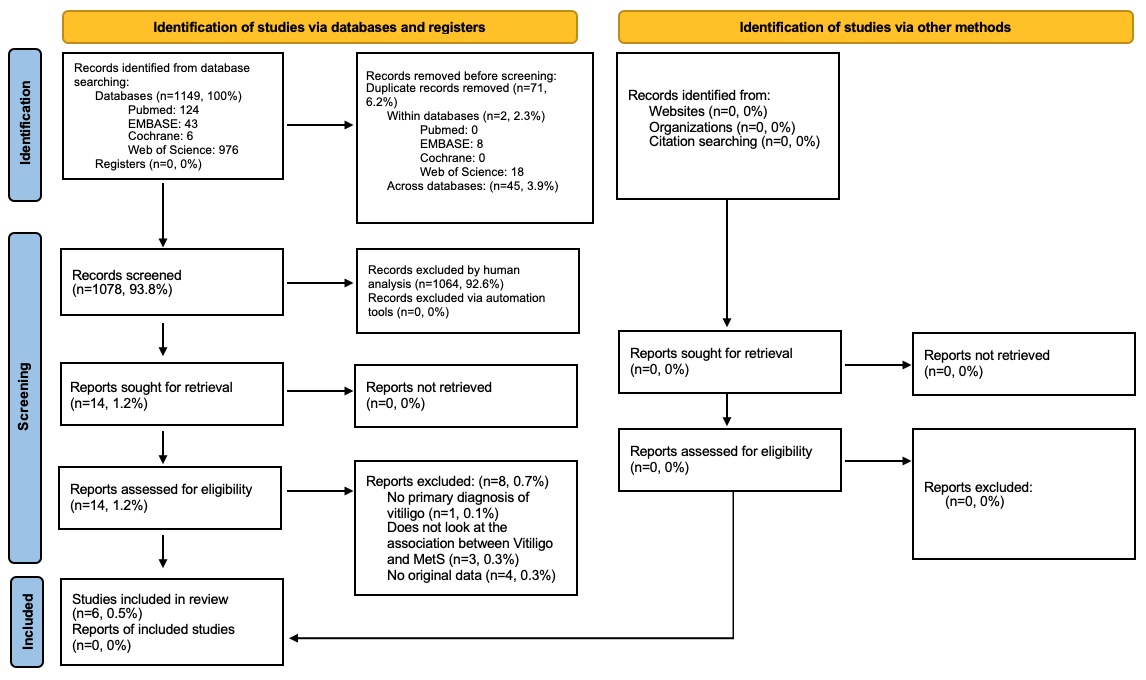
PRISMA (Preferred Reporting Items for Systematic Reviews and Meta-Analyses) study flowchart. MetS: metabolic syndrome.

This study was conducted in accordance with PRISMA (Preferred Reporting Items for Systematic Reviews and Meta-Analyses) guidelines [23] and is illustrated in Figure 1.

### Literature Search

A comprehensive literature search of the electronic databases Pubmed, Embase, Cochrane, and Web of Science was carried out for all published literature from inception through August 16, 2020. The search terms used were found within the title, abstract, full text, or keywords. Search words included “vitiligo,” “leukoderma,” “metabolic syndrome X,” “dysmetabolic syndrome X,” “insulin resistance syndrome X,” and “syndrome X” (Supplemental Table 1 in Multimedia Appendix 1). The conjunctions “AND” and “OR” were used to yield maximal results. Additionally, a manual search of each included study’s reference list was performed to identify other relevant papers. No geographic or temporal restrictions were imposed. No gray literature was searched or included in the review, neither were dissertations, books, letters to the editor, or unpublished studies.

### Study Selection

All studies were screened by 2 independent reviewers (JX and CM), and disagreements were resolved via a third independent party (WG). Of the papers produced by our search, the titles and abstracts were reviewed for eligibility. Papers that were deemed irrelevant based on title and abstract alone were not further analyzed, whereas those that were deemed relevant went on to full text review. Studies meeting any of the exclusion criteria were retracted from further analyses.

### Inclusion Criteria

The inclusion criteria for this study were as follows: (1) only published articles written in English language from inception to August 16, 2020; (2) observational studies examining the association of vitiligo with MetS, including cross-sectional, case-control, or cohort studies; (3) studies that diagnosed subjects with MetS based on either NCEP ATP III [24,25], IDF [26], or Harmonization [10] criteria and specifically analyzed the relationship between vitiligo and all components of MetS. Studies discussing all forms of vitiligo were eligible for inclusion. No specific duration of vitiligo of MetS from diagnosis was necessary for inclusion; and (4) studies containing control groups n≥5.

### Exclusion Criteria

The exclusion criteria for this study were as follows: (1) studies that did not specifically examine all components of MetS (eg, those only analyzing the relationship between vitiligo and insulin resistance or vitiligo and blood pressure); (2) studies using nonhuman subjects; (3) papers not written in English; (4) papers for which full text was not available; and (5) papers in the format of dissertations, books, or letters to the editor.

### Data Extraction and Risk of Bias Assessment

Data extracted from the included studies consisted of first author, year of publication, country and city of origin, study type, total sample size, case group size, control group size, mean age, percentage of female participants, type of vitiligo, diagnostic criteria for vitiligo, inclusion criteria for vitiligo cases, percentage of affected body surface area, mean vitiligo disease duration, inclusion criteria for controls, number of patients diagnosed with MetS, MetS criteria for diagnosis, reported odds ratio (95% CI) for development of MetS in patients with vitiligo, MetS component values, fasting glycemic index (FGI), triglycerides, high-density lipoprotein (HDL) cholesterol, systolic blood pressure (SBP), diastolic blood pressure (DBP), waist circumference, low-density lipoprotein (LDL) cholesterol, BMI, smoking status, and alcohol use status (Supplemental Table 2 in Multimedia Appendix 1) [19,20,27-30].

We used the Newcastle-Ottawa Scale (NOS) to assess risk of bias (Figure 1A [19,20,27-29] and 1B [30] in Multimedia Appendix 1). Separate scales were used to rate case control papers and cross-sectional papers. Case control papers were rated with regard to adequate definition of cases, representativeness of sample, representativeness of controls, definition of controls, comparability of cases and controls based on age and sex, adequacy of ascertainment of exposure, comparability of ascertainment method across cases and controls, and nonresponse rate. Cross-sectional papers were rated on an adapted scale for representativeness of sample, sample size, nonresponse rate, method of ascertainment of exposure, comparability of samples based on age and sex, method of outcome assessment, and viability of statistical analysis used. Two authors (CM and JX) individually scored each paper on these scales with a third author (WG) weighing in as a tiebreaker. We considered an NOS score greater than or equal to 5/9 as low risk of bias.

### Statistical Analysis

A pooled odds ratio on the association between vitiligo and MetS and all mean differences for subgroup analyses were calculated and depicted in forest plots using Review Manager (version 5.4, Cochrane Collaboration) [31]. A random effects model of Mantel-Haenszel was used for the odds ratio due to high heterogeneity, as determined by I^2 values greater than 50%. Calculations for mean differences used an inverse variance method with a random effects or fixed effects model as determined by I^2 degree of heterogeneity. Pooled prevalence of MetS in patients with vitiligo was conducted using OpenMeta[Analyst], version 10.2 [32], using the random effects models of DerSimonian-Laird. All calculations were performed with a 95% CI. *P* values of <.05 were considered significant.

## Results

### Characteristics of Included Studies

Our search identified 1149 records by title alone. After duplicates were removed, 1078 records were reviewed for applicability. Of these records, 1064 articles were excluded based on title and abstract screening. The remaining 14 articles underwent full text review to assess for eligibility, 6 of which met the inclusion criteria. A summary of the inclusion process is presented in Figure 1. The characteristics of the included studies are listed in Supplemental Table 3 in Multimedia Appendix 1 [19,20,27-30]. Five papers were case control studies [19,20,27-29], and 1 was a prospective cross-sectional study [30]. Moreover, 3 studies were conducted in India [27-29], 2 in Turkey [19,30], and 1 in Egypt [20]. A total number of 734 participants (375 of which were diagnosed with vitiligo) were included across all studies: 128 (63 with vitiligo, 49.2%) from Atas et al [19], 191 (102 with vitiligo, 53.4%) from Sallam et al [20], 200 (100 with vitiligo, 50%) from Sharma et al [27], 65 (35 with vitiligo, 53.8%) from Singh et al [28], 150 (75 with vitiligo, 50%) from Sinha et al [29], and 310 (155 with vitiligo, 50%) from Tanacan et al [30]. The type of vitiligo varied across papers, with both segmental and nonsegmental types examined in 3 studies [19,20,30]; 1 paper exclusively studied nonsegmental types [27], and 2 studies did not specify the type of vitiligo the patients were diagnosed with [28,29]; 3 studies reported the duration of vitiligo (in years): 9.5 (SD 8.1) [19], 5.29 (SD 6.8) [20], and 43.5 (SD 10.5) [27]; however, the duration was statistically significant across these studies (*P*=.03). The diagnostic criteria for MetS also varied among studies, with 4 studies using NCEP ATP III criteria [19,27,29,30] and 2 using IDF criteria [20,28]. Two studies [27,30] took into consideration social risk factors such as alcohol and smoking use; Sharma et al [27] report no significant association between smoking (*P*=.31) or alcohol (*P*=.28) and the development of MetS in patients with vitiligo. Tanacan et al [30] report no significant relationship (*P*=.81) regarding smoking, but a significant relationship was observed (*P*=.01) regarding alcohol consumption. Comorbid conditions were not examined in any of the studies included.

### Risk of Bias of the Included Studies

The risk of bias of the included studies is summarized in Supplemental Figure 1A [19,20,27-29] and 1B in Multimedia Appendix 1 [30]. The NOS was used to assess bias in the 5 case control studies [19,20,27-29], with a modified NOS scale adapted for cross-sectional studies [30]. Except for Sinha et al [29], all included studies [19,20,27,28,30] were rated at low risk of bias (ie, NOS score greater than or equal to 5). We rated Sinha et al [29] at high risk of bias because the same method of ascertainment for cases and controls was not used. The reason for unclear risk of bias in the nonresponse rate domain by Sinha et al was due to a discrepancy in the sample size for the control group without mention of loss to follow-up.

### Prevalence and Association of Vitiligo With Metabolic Syndrome

**Figure 2:**
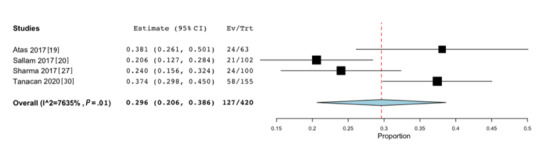
Forest plot of the pooled prevalence of metabolic syndrome in patients with vitiligo (*P*<.001). Ev/Trt: number of events in experimental/treated group.

**Figure 3:**
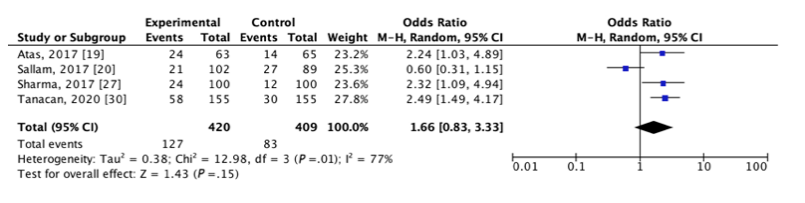
Forest plot of the association of vitiligo with metabolic syndrome: odds of vitiligo patients developing metabolic syndrome compared to healthy control. M-H: Mantel-Haenszel.

Four studies presented the necessary data to determine the pooled prevalence of MetS in patients with vitiligo. Due to the high heterogeneity (I^2^=76%), a random effects model of DerSimonian-Laird was adopted for the calculations. We calculated a pooled prevalence of 29.6% (95% CI, 20.6%-38.6%; *P*<.001; Figure 2) [19,20,27,30]. Individual studies had a prevalence ranging from 20.6% to 38.1%. These same 4 studies [19,20,27,30] were used to calculate the odds ratio. Overall, patients with vitiligo were not more likely to develop MetS compared to age-matched and sex-matched control patients (odds ratio 1.66, 95% CI 0.83, 3.33; *P*=.01; Figure 3 [19,20,27,30]). However, sensitivity analysis with removal of one study at a time revealed a statistically significant association between vitiligo and MetS when Sallam et al [20] was removed (odds ratio 2.39, 95% CI 1.64, 3.47; *P*<.001). Substantial statistical heterogeneity was reported across these 4 studies (I^2^=77%).

### Components of Metabolic Syndrome in Patients With Vitiligo

**Figure 4:**
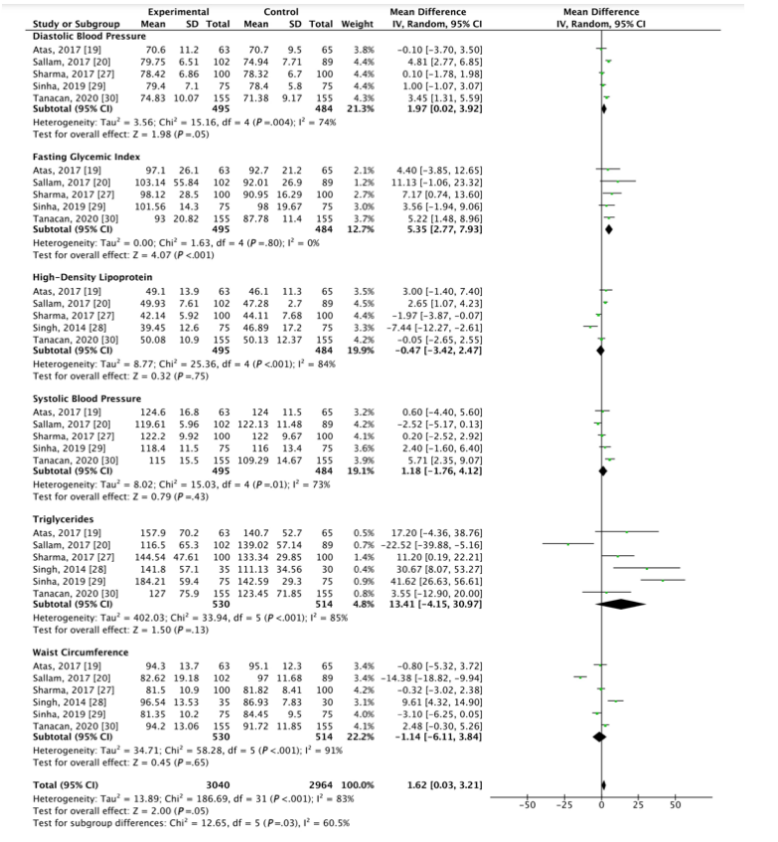
Forest plots of the mean difference of vitiligo with the components of metabolic syndrome.

A minimum of 5 studies [19,20,27-30] were used to calculate the mean difference of waist circumference, triglycerides, HDL, SBP, DBP, and FGI between vitiligo and control groups; significant elevations in FGI (mean difference [MD] 5.35, 95% CI 2.77, 7.93; *P*<.001) and DBP (MD 1.97, 95% CI 0.02, 3.92; *P*=.05) were observed in patients with vitiligo compared to age-matched and sex-matched control patients (Figure 4 [19,20,27-30]). Substantial statistical heterogeneity was found in DBP (I^2^=74%), but not in FGI (I^2^=0%). No significant difference was observed between patients with vitiligo and control patients regarding waist circumference (MD -1.14, 95% CI -6.11, 3.84; *P*<.001), HDL cholesterol (MD -0.47, 95% CI -3.42, 2.47; *P*<.001), SBP (MD 1.18, 95% CI -1.76, 4.12; *P*<.01), or triglycerides (MD 13.42, 95% CI -4.13, 30.97; *P*<.001). A leave-one-out sensitivity analysis revealed a significant elevation in triglyceride levels with removal of Sallam et al (MD 20.44, 95% CI 6.07, 34.81; *P*=.01; Supplemental Figure 2 in Multimedia Appendix 1 [19,20,27,30]). No significant changes were detected with sensitivity analysis across the remaining MetS components.

### Additional Metabolic Measurements in Patients With Vitiligo

**Figure 5:**
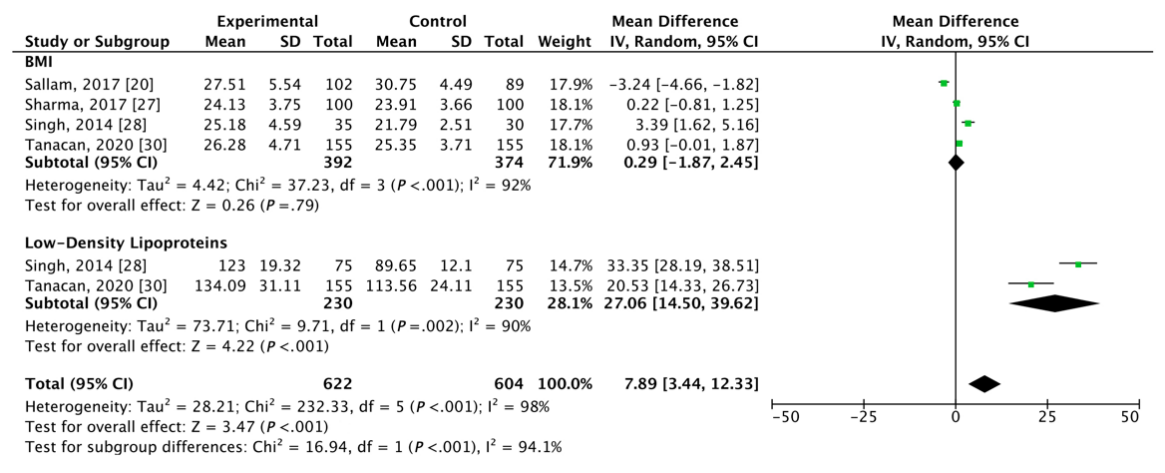
Forest plots of the mean difference of vitiligo with additional metabolic changes (low-density lipoprotein cholesterol and BMI).

Figure 5 [20,27,28,30] depicts the mean differences between patients with vitiligo and control patients regarding LDL cholesterol and BMI. Two studies [28,30] were used to calculate the mean difference in LDL cholesterol. A significant elevation in mean LDL cholesterol levels was reported in patients with vitiligo as compared to age-matched and sex-matched control patients (MD 27.06, 95% CI 14.50, 39.62; *P*<.001) with substantial heterogeneity identified across both studies (I^2^=90%). Four studies [20,27,28,30] were used to calculate the mean difference of BMI between patients with vitiligo and control patients; however, no significant difference was detected even after sensitivity analyses (MD 0.29, 95% CI -1.87, 2.45; *P*<.001). Statistically significant heterogeneity was identified across all 4 studies (I^2^=92%).

## Discussion

### Analysis

The recommendation for metabolic screening in patients with vitiligo has not been well defined. While previous literature suggests a shared pathophysiology between vitiligo and metabolic syndrome (MetS), the association between the 2 conditions remains unclear. In our study, we approximate the prevalence of MetS in patients with vitiligo to be about 30%, corroborating rates of MetS seen in the general population. A 2017 study by Moore et al [33] found that the prevalence of MetS among US adults aged 18 years and older was approximately 34.2% from the period of 2007-2012, while a 2018 paper by Saklayen [9] estimates the global MetS prevalence to be approximately 25%. While the prevalence of MetS in patients with vitiligo is similar to that of the general population, we still recommend increased vigilance in patients with vitiligo due to the perceived risk for cardiovascular complications that may result from MetS.

While 5 [19,27-30] of the 6 research articles analyzed in this review demonstrate a significant association between vitiligo and MetS, our study shows an overall lack of association between vitiligo and MetS; however, a leave-one-out sensitivity analysis removing Sallam et al reveals that a significant association does exist [19,20,27,30]. Leave-one-out analyses are commonly performed to isolate studies that have disproportionate effect sizes on the overall meta-analysis. With exclusion of Sallam et al [20] producing a significant change in the results, consideration must be given as to whether the study is an outlier. It is possible that the nonsignificant findings observed in this study may be explained by the relatively short duration of vitiligo (2-6 years) among diagnosed cases [20]. Shorter vitiligo duration may allow less time for the development of MetS, possibly skewing the results.

A closer look at the diagnostic components of MetS demonstrates a significantly higher FGI in patients with vitiligo when compared to age-matched and gender-matched controls, though the mean for both groups remained within normal range (FGI of 96.66 in patients with vitiligo vs 91.30 in controls). The increased FGI seen in the vitiligo group brings this group closer to the prediabetes threshold of a value greater than 100. Several studies have reported an increased incidence of vitiligo as a result of insulin resistance [16]. It is possible that the elevation in FGI observed in patients with vitiligo reflect early changes of insulin resistance that may eventually progress to metabolic disease. While there are no current guidelines regarding yearly hemoglobin A1C screening for patients with vitiligo, these findings suggest a potential benefit in early glucose monitoring in patients diagnosed with vitiligo.

LDL cholesterol levels and BMI are outside of the diagnostic criteria for MetS. However, a case control study by Houssien et al [34] showed an increased incidence of chronic diseases such as type 2 diabetes, dyslipidemia, and obesity in patients with vitiligo. Consistent with the literature, we found a significant elevation in mean LDL cholesterol levels in patients with vitiligo compared to control groups. Similar to the elevations in FGI, patients with vitiligo had elevated LDL cholesterol levels, which may suggest an increased predisposition for metabolic derangements. On the other hand, no significant difference in mean BMI was observed across groups even after sensitivity analysis, suggesting that obesity may not be the underlying mechanism for metabolic disturbances observed in patients with vitiligo [16].

Alterations in cytokine production, autoimmunity, and genetic predisposition are thought to be the main factors in the pathogenesis of vitiligo [30]. Increased levels of proinflammatory cytokines such as TNF-α IL-1, and IL-6 have been shown to promote insulin resistance and cause metabolic disturbances in children with vitiligo [7]. Additionally, there is evidence that melanin exerts anti-inflammatory and antioxidant effects in adipose tissue [35]; thus, the decreased number of melanocytes and decreased melanogenesis seen in patients with vitiligo could serve as a source of oxidative stress involved in the pathogenesis of MetS [7]. Finally, homocysteine levels have been noted to be increased in patients with vitiligo as compared to control groups [36]. This molecule inhibits tyrosinase in melanin synthesis, acting as another potential contributor to vitiligo pathogenesis; in fact, elevated levels are a known risk factor for cardiovascular disease [36]. Such inflammatory changes are important to consider when assessing the risk of MetS in patients with vitiligo.

Interestingly, certain treatments for vitiligo have demonstrated cardiovascular benefits as well. A study by Bae et al [37] noted significantly decreased risk of subsequent cardiovascular and cerebrovascular events in patients with vitiligo who were treated with narrowband UV-B phototherapy when compared to the untreated group. The 2 groups were matched for covariables such as diabetes, hypertension, and hyperlipidemia, though the effects of treatment on these factors was not reported. While it is unclear as to whether this improvement was an effect of the treatment of vitiligo or UV-B therapy in and of itself, this finding emphasizes the need for further research regarding the effects of other common vitiligo therapies, such as topical steroids, on the prevention of cardiovascular disease.

### Limitations

There are several limitations of this study. First, a small number of studies were included due to the paucity of literature on vitiligo and metabolic syndrome. There is a need for more comprehensive studies with a larger sample size. Second, though most papers reported study populations with a mean age corresponding to an adult cohort, Sinha et al [29] specified only that the study population was over 18 years in age. Therefore, though our findings largely apply to an adult population, we cannot exclude the possibility that geriatric patients were included in analysis. Our papers also did not report on the racial breakdown of the study groups. We therefore cannot exclude race as a confounder, and do not know the extent to which race affects access to medical care in the study countries. Third, except for Sallam et al [20], the criteria for diagnosing vitiligo were not specified, and different subtypes of vitiligo were evaluated across studies. While some studies included patients with both segmental and nonsegmental vitiligo [19,20,30], others limited their studies to include only nonsegmental vitiligo cases [27], and 2 studies did not specify [28,29]. Because nonsegmental vitiligo has been associated more with chronic inflammation and MetS as compared to segmental vitiligo [30], it is important to differentiate which subtypes are under investigation. Lastly, there were 3 diagnostic criteria used in this study for identifying MetS in patients with vitiligo, which were NCEP, IDF, and Harmonization guidelines. Although the guidelines differ only regarding waist circumference, a more consistent approach to diagnosing MetS should be used in the future. Future studies should examine the impact of other factors such as age, gender, race, and duration or severity of vitiligo in the development of MetS.

### Conclusions

The association between vitiligo and metabolic syndrome carries important clinical implications that warrant increased vigilance by dermatologists and other health care professionals involved in the care of this unique patient population. Surveillance of FGI and LDL cholesterol levels may be beneficial in reducing serious cardiovascular complications that may result from comorbid metabolic disease. Further studies are needed to determine the extent of cardiometabolic derangements in order to set guidelines for monitoring and preventing disease progression.

## Appendix

Multimedia Appendix 1 Supplementary figures and tables.

## Abbreviations

ATP: Adult Treatment Panel
DBP: diastolic blood pressure
FGI: fasting glycemic index
HDL: high-density lipoprotein
IDF: International Diabetes Federation
IFN-γ: interferon gamma
IL: interleukin
LDL: low-density lipoprotein
MD: mean difference
MetS: metabolic syndrome
NCEP: National Cholesterol Education Program
PRISMA: Preferred Reporting Items for Systematic Reviews and Meta-Analyses
SBP: systolic blood pressure
TNF-α: tumor necrosis factor alpha
