# Supplementary material for: Vitiligo and Metabolic Syndrome: Systematic Review and Meta-Analysis
Source: JMIR Dermatol. 2022 Mar 16;5(1):e34772. doi: 10.2196/34772 (PMC10334901; doi:10.2196/34772)
Supplement: Multimedia Appendix 1 [file derma_v5i1e34772_app1.docx]

**Supplementary Materials:**

**Supplemental Figure 1A. Detailed Newcastle-Ottawa Scale of each included case control study**

**
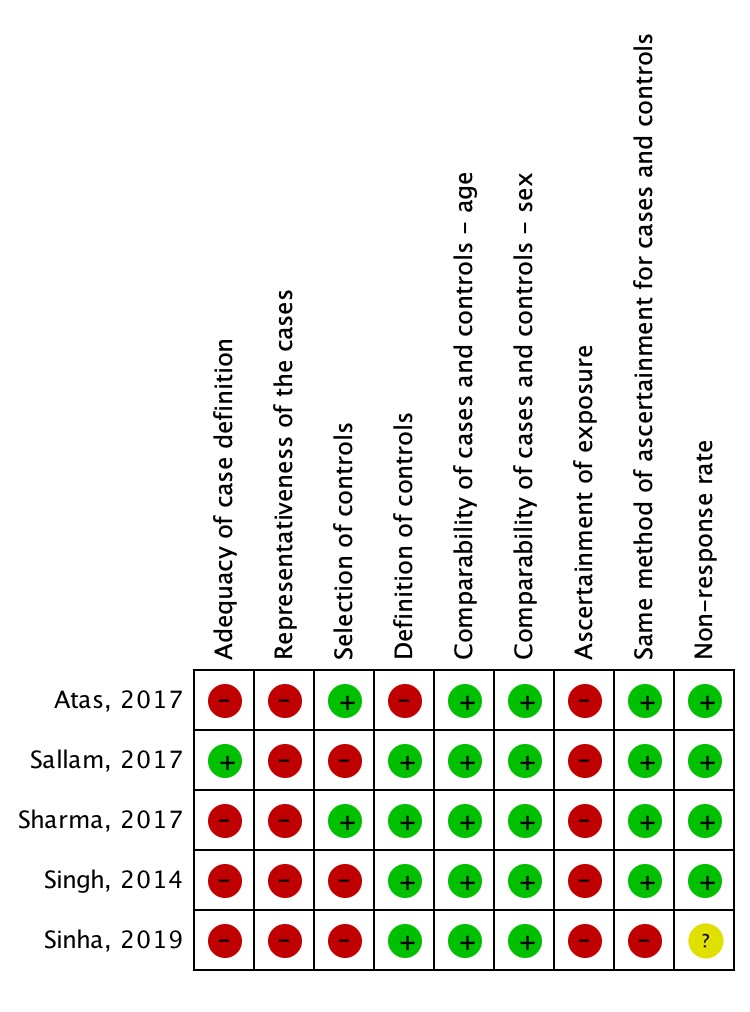
**

**Supplemental Figure 1B. Detailed Newcastle-Ottawa Scale of each included cross-sectional study**

**
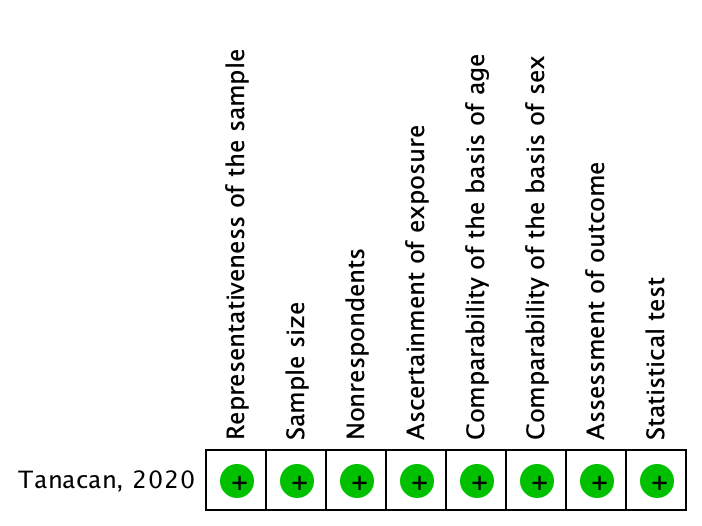
**

**Supplemental Figure 2. Sensitivity Analysis for the Odds of Vitiligo Patients Developing Metabolic Syndrome Compared to Healthy Control**

**Sensitivity Analysis**

| **Study Removed** | **OR** | **P Value** |
| --- | --- | --- |
| **Atas, 2017** | 1.52 [0.61, 3.80] | 0.37 |
| **Sallam, 2017** | 2.39 [1.64, 3.47] | <0.00001 |
| **Sharma, 2017** | 1.50 [0.60, 3.76] | 0.39 |
| **Tanacan, 2020** | 1.43 [0.57, 3.59] | 0.45 |

| **Supplemental Table 1. Search terms** |
| --- |
| **PubMed MEDLINE : August 16, 2020. 124 results.**  **After deduplication on EndNote on August 16, 2020. 124 results**     1. **“Vitiligo” [Mesh]** 2. **“Leukoderma” [Mesh]** 3. **Vitiligo** 4. **Leukoderma** 5. **Leucoderma** 6. **1 or 2 or 3 or 4 or 5** 7. **“Metabolic syndrome X” [Mesh]** 8. **“Insulin resistance syndrome X” [Mesh]** 9. **“Dysmetabolic syndrome X [Mesh]** 10. **Metabolic syndrome X** 11. **Metabolic syndrome** 12. **Insulin resistance syndrome X** 13. **Insulin resistance syndrome** 14. **Dysmetabolic syndrome X** 15. **Dysmetabolic syndrome** 16. **Syndrome X** 17. **7 or 8 or 9 or 10 or 11 or 12 or 13 or 14 or 15 or 16** 18. **6 and 17** |
| **EMBASE - August 16, 2020. 43 results.**  **After deduplication on EndNote on August 16, 2020. 35 results**    **1. ‘vitiligo’/exp**  **2. ‘vitiligo’**  **3. ‘leukoderma’/exp**  **4. ‘leukoderma’**  **5. ‘leucoderma’**  **6. #1 OR #2 OR #3 OR #4 OR #5**  **7. ‘metabolic syndrome X’/exp**  **8. ‘metabolic syndrome X’**  **9. 'metabolic syndrome'**  **10.  ‘insulin resistance syndrome x’**  **11.  'insulin resistance syndrome'**  **12.  'dysmetabolic syndrome x'**  **13.  ‘dysmetabolic syndrome’**  **14.  'syndrome x'/exp**  **15.  ‘syndrome x’**  **16.  #7 OR #8 OR #9 OR #10 OR #11 OR #12 OR #12 OR #14 OR #15**  **17.  #6 AND #16** |
| **COCHRANE CENTRAL: August 16, 2020. 6 results.**  **After deduplication on EndNote on August 16, 2020. 6 results**    **1. Vitiligo**  **2. MeSH descriptor: [Vitiligo] 1 tree(s) exploded**  **3. leukoderma**  **4. leucoderma**  **5. #1 OR #2 OR #3 OR #4**  **6. Metabolic syndrome**  **7. MeSH descriptor: [Metabolic Syndrome] explode all trees**  **8. metabolic syndrome x**  **9. dysmetabolic syndrome x**  **10.  dysmetabolic syndrome**  **11.  insulin resistance syndrome x**  **12.  insulin resistance syndrome**  **13.  syndrome x**  **14.  #6 OR #7 OR #8 OR #9 OR #10 OR #11 OR #12 OR #13**  **15.  #5 AND #14** |
| **WEB OF SCIENCE: August 16, 2020. 976 results.**  **After deduplication on EndNote on August 16, 2020. 958 results**    **1. Vitiligo**  **2. Leukoderma**  **3. Leucoderma**  **4. #1 or #2 or #3**  **5. Metabolic Syndrome X**  **6. Metabolic Syndrome**  **7. Dysmetabolic syndrome X**  **8. Dysmetabolic syndrome**  **9. Insulin resistance syndrome X**  **10.  Insulin resistance syndrome**  **11.  Syndrome X**  **12.  #5 or #6 or #7 or #8 or #9 or #10 or #11**  **13.  4 and 12** |

**Supplemental Table 2: Complete Data Extraction Table**

**
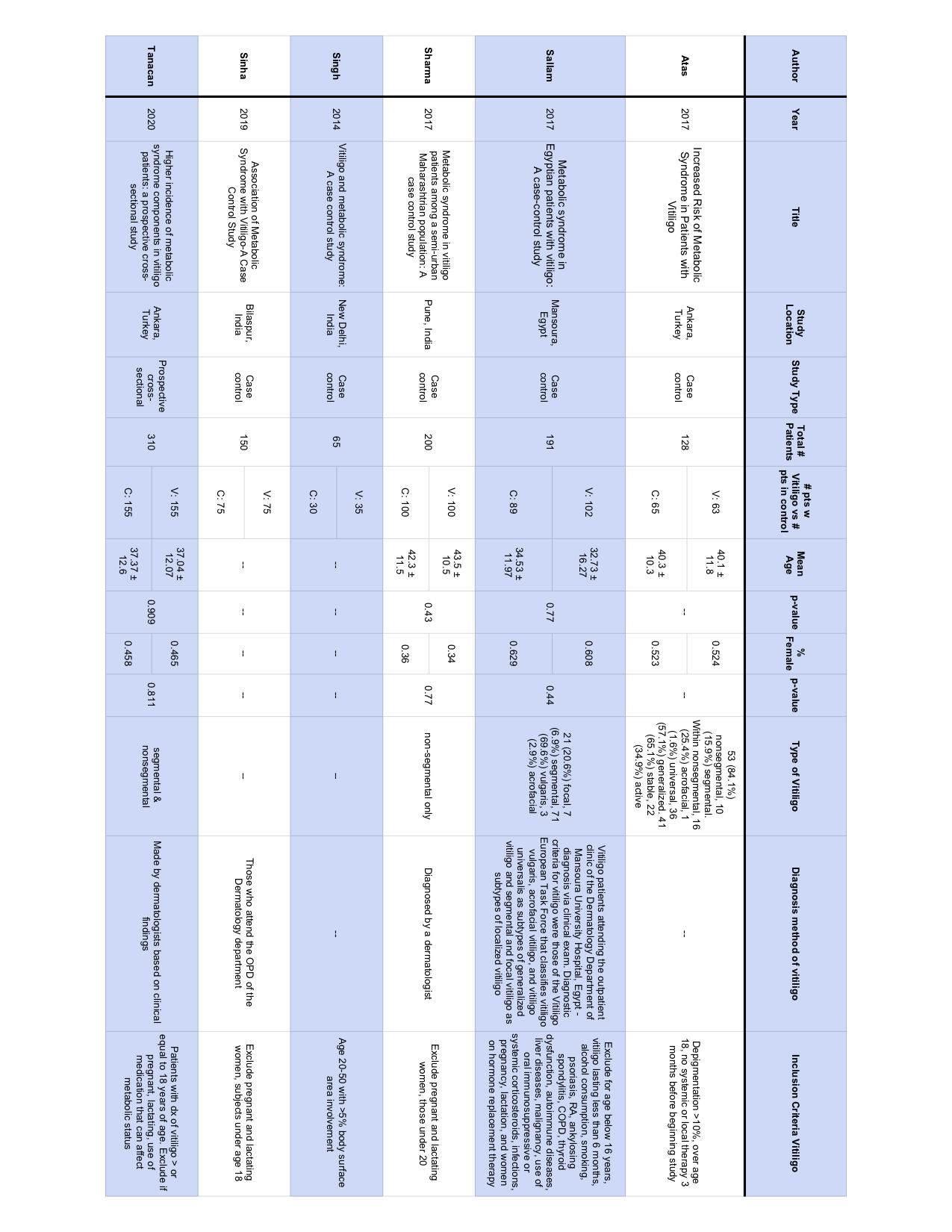
**

**
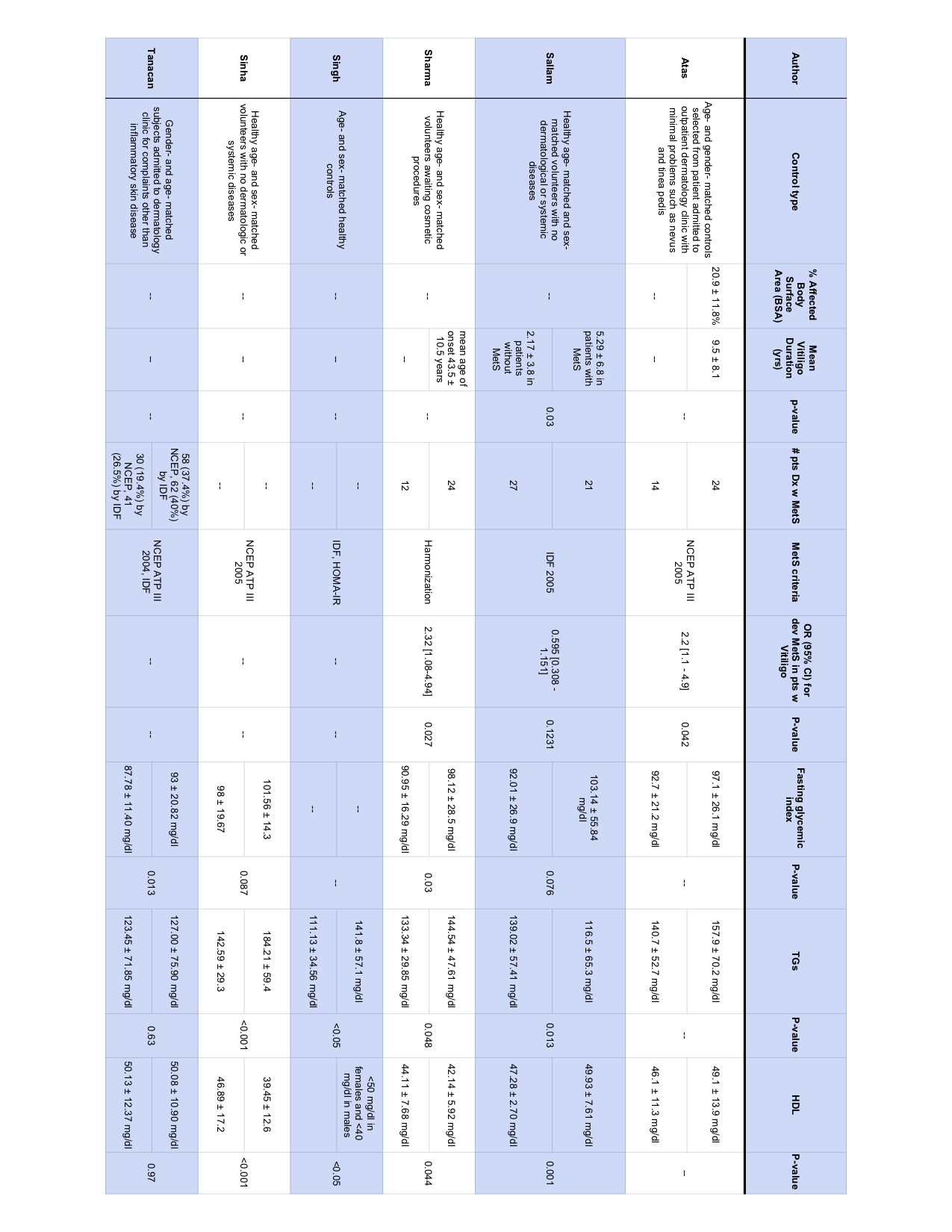
**

**
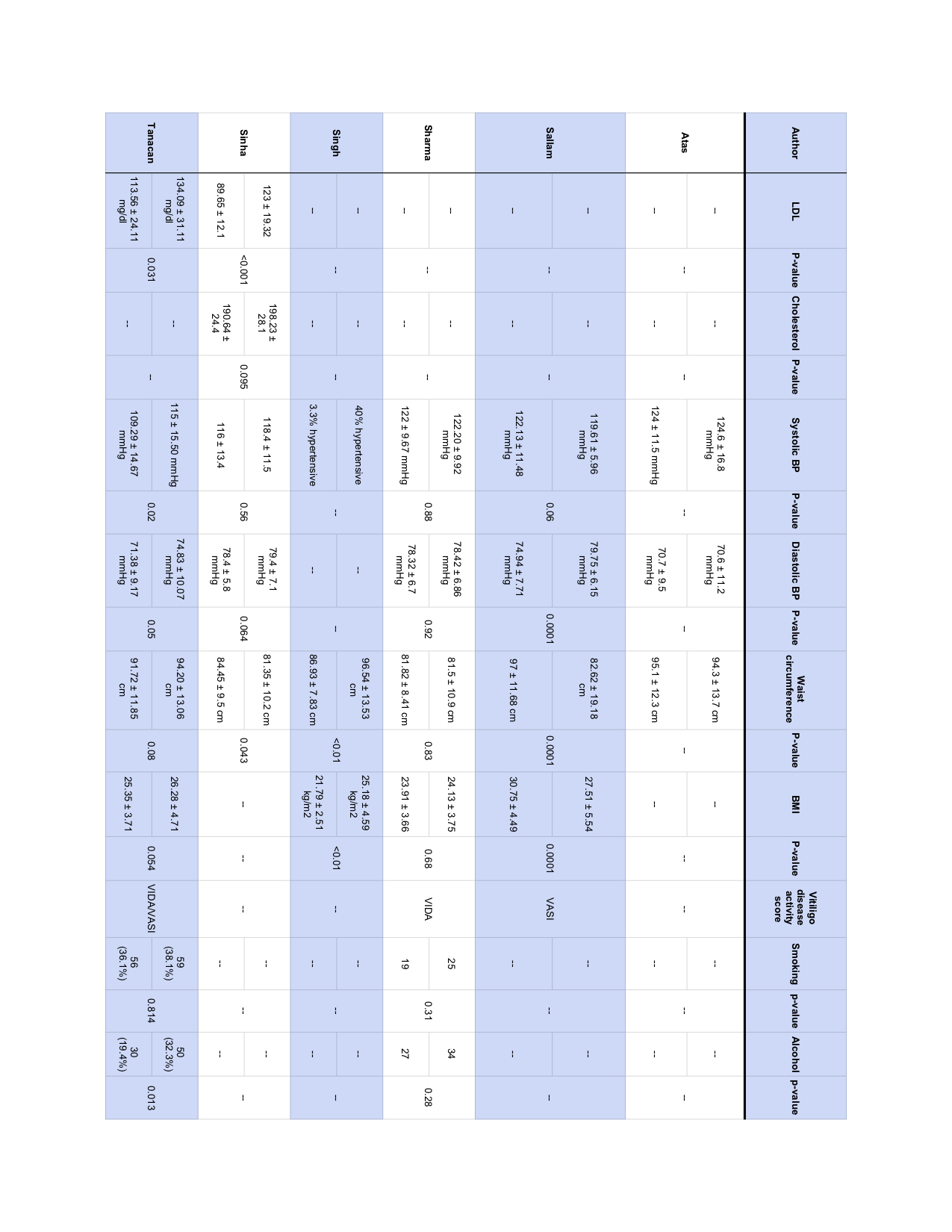
**

**Supplemental Table 3. Characteristics of Included Studies**

|  |  | **Sample Size n**  **(% female)** | |  | **# pts dx w MetS** | |  |  |
| --- | --- | --- | --- | --- | --- | --- | --- | --- |
| **Source** | **Study Design** | **Vitiligo** | **Control** | **MetS Diagnostic Criteria** | **Vitiligo** | **Control** | **OR (95% CI) MetS in vitiligo pts** | **Components used** |
| Atas, 2017 | Case control | 63 (52) | 65 (52) | NCEP ATP III | 24 | 14 | 2.2 [1.1 - 4.9] | Waist circumference, TAGs, HDL, SBP, DBP, FGI |
| Sallam, 2017 | Case control | 102 (33) | 89 (35) | IDF | 21 | 27 | NA | Waist circumference, TAGs, HDL, SBP, DBP, FGI, BMI |
| Sharma, 2017 | Case control | 100 (44) | 100 (42) | Harmonization | 24 | 12 | 2.32 (1.08-4.94) | Waist circumference, TAGs, HDL, SBP, DBP, FGI, BMI |
| Singh, 2014 | Case control | 35 | 30 | IDF | NA | NA | NA | Waist circumference, TAGs, BMI |
| Sinha, 2019 | Case control | 75 | 75 | NCEP ATP III | NA | NA | NA | Waist circumference, TAGs, HDL, LDL, SBP, DBP, FGI |
| Tanacan, 2020 | Prospective cross-sectional | 155 (37) | 155 (37) | NCEP ATP III + IDF | 58 (NCEP)  62 (IDF) | 30 (NCEP)  41 (IDF) | NA | Waist circumference, TAGs, HDL, LDL, SBP, DBP, FGI, BMI |
